# Differentially induced immunity in buccal and nasal mucosae after vaccination for SARS–CoV–2: Prospects for mass scale immunity-screening in large populations

**DOI:** 10.3389/fimmu.2022.999693

**Published:** 2022-11-17

**Authors:** Chrysanthi Tsamadou, Carolin Ludwig, Judith Scholz, Matthias Proffen, Janina Hägele, Immanuel Rode, Sixten Körper, Dorit Fabricius, Bernd Jahrsdörfer, Christine Neuchel, Elisa Amann, Hubert Schrezenmeier, Daniel Fürst

**Affiliations:** ^1^ Institute of Clinical Transfusion Medicine and Immunogenetics Ulm, German Red Cross Blood Transfusion Service, University Hospital Ulm, Ulm, Baden-Wuerttemberg, Germany; ^2^ Institute of Transfusion Medicine, University of Ulm, Ulm, Germany; ^3^ Department of Pediatrics and Adolescent Medicine, Ulm University Medical Center, Ulm, Germany

**Keywords:** SARS-CoV-2, vaccination, buccal mucosa, nasal mucosa, immunity

## Abstract

**Introduction:**

Humoral immunity after SARS-CoV-2 vaccination has been extensively investigated in blood. Aim of this study was to develop an ELISA method in order to determine the prevalence of IgG and IgA SARS-CoV-2 domain 1 spike-protein (S) specific antibodies (Abs) in buccal and nasal mucosal surfaces of vaccinees.

**Methods:**

To this end, we analyzed 69 individuals who received their first vaccine dose between February and July 2021. Vaccines administered were BNT162b2, mRNA-1273 or ChAdOx1-nCoV-19. Detection of IgG and IgA Abs was performed using commercial ELISA kits for both blood and swab samples after protocol modification for the latter.

**Results:**

Anti-spike IgG and IgA Abs in the buccal and/or nasal swabs were detectable in >81% of the study subjects after the second dose. The IgG measurements in buccal swabs appeared to correlate in a more consistent way with the respective measurements in blood with a correlation coefficient of r=0.74. It is of note that IgA Abs appeared to be significantly more prevalent in the nasal compared to the buccal mucosa. Optimal selection of the assay cut-off for the IgG antibody detection in buccal swabs conferred a sensitivity of 91.8% and a specificity of 100%. Last, individuals vaccinated with mRNA-based vaccines exhibited higher antibody levels in both blood and mucosal surfaces compared to those receiving ChAdOx1-nCoV-19 confirming previously reported results.

**Conclusion:**

In conclusion, our findings show a differential prevalence of anti-S Abs on mucosal surfaces after vaccination for SARS-CoV-2, while they also set the basis for potential future use of IgG antibody detection in buccal swabs for extended immunity screening in large populations.

## Introduction

The emergence of SARS-CoV-2 has challenged the world during the last two and a half years in an unprecedented scale with more than 528,000,000 confirmed recorded cases and more than 6,000,000 deaths (1). Between the first report of a pneumonia outbreak of unknown origin in the Chinese city of Wuhan around December 2019 and the third quarter of 2020, the scientific community raced against time in order to decipher the newly emerged pathogen and to develop efficient vaccines against it (2). Indeed, in record time vaccines based on new (i.e. mRNA, viral vector, DNA) or more traditional (i.e. inactivated virus, subunit) technology were developed (3–5), validated and received emergency authorization for use in big parts of the world (6). A plethora of scientific reports has since then provided a significant insight into the efficacy and safety profile of these vaccines (7). However, although the post-vaccination immunological response has been extensively investigated in blood, far less is known about the mounted immunity at the mucosal surfaces of the upper respiratory system, which also constitute the main route of entry of the virus (8, 9). Secretory IgA antibodies (Abs) are the main but not exclusive component of mucosal humoral immunity, as IgG Abs either plasma-derived or locally produced are also prevalent on mucosal surfaces (10). Although the ability of these mucosa-prevalent pathogen-specific IgG Abs to inhibit infection is generally underestimated (11), it has been reported that they may play an important role in mucosal protection against HIV transmission (12). Primary objective of this study was the development of a valid immunoassay for detection of IgG and IgA Abs against the S1 domain of the SARS-COV-2 spike (S) protein in swab samples from the buccal and nasal mucosae. Further aim was to assess and compare the ability of the first three European Medicines Agency (EMA)-authorized vaccines for SARS-CoV-2, namely BNT162b2 by Pfizer-BioNTech, mRNA-1273 by Moderna and ChAdOx1-nCoV-19 by AstraZeneca, to induce a measurable mucosal humoral immunity. To this end, anti-S protein specific IgG and IgA Abs were measured using a semi-quantitative ELISA assay in serum, buccal as well as nasal swab samples from healthy individuals who received their first vaccine dose between February and July 2021.

## Methods

### Study design and participants

For this observational cohort study we recruited and analyzed 69 adult individuals who underwent a full SARS-CoV-2 vaccination with BNT162b2, mRNA-1273, ChAdOx1-nCoV-19 or combination between February and July 2021. Blood and swab samples (i.e. buccal and nasal) were collected before and after each vaccination dose in order to determine the prevalence of IgG and IgA anti-S Abs in both serum and mucosal surfaces. The clinical protocol for sample and data collection along with the informed written consent were approved by the Institutional Review Board at Ulm University (No. 488/20).

Severe immunodeficiency, pregnancy and intake of glucocorticosteroids were predefined as exclusion criteria. All participants were older than 18 years and were eligible to receive vaccination against SARS-CoV-2. The administered vaccination schemes adhered to the then in force recommendations of the German Standing Committee on Vaccination (STIKO) and as previously described (13).

The blood and swab samples were collected up to one week before or on day of first vaccination, two to four weeks after first vaccine dose depending on vaccination scheme (i.e. BNT162b2, mRNA-1273, ChAdOx1-nCoV-19) and one to four weeks after second vaccine dose. Sample collection was continued on a three-month interval basis after second vaccine dose up to one year after first vaccination dose. These measurements were, however, not included in this analysis due to the emergence of multiple vaccination schemes with respect to the booster vaccine doses administered. The blood samples were collected in two 5 ml or one 8 ml container with gel separator. After centrifugation at 3000 rpm for 10-15 minutes, serum was efficiently separated from the rest blood components at the upper compartment of the container. The swab samples were self-collected by the participants after sufficient training and supervision of trained personnel. Swabs with flocking technology on tips (i.e. flocked swabs) from Copan Italia S.p.A. were used for sample collection in mouth and nose as this kind of swabs have been shown to ensure better sample yield and more efficient release of sample into liquid media compared to non-flocked swabs (14).

Following clinical data were recorded throughout the study: age, sex, date and type of vaccine administered, previous or breakthrough infection with SARS-CoV-2 confirmed by nucleic acid amplification test (NAAT) with additional information on diagnosis date and disease course as well as prevalence and intensity of side-effects after vaccination. The latter were categorized according to intensity as non-existing, mild or moderate. No severe side-effects that would require hospitalization were observed in any of the participants. As mild were considered local reactions at injection spot or mild systemic reactions like for example mild fatigue and headache. As moderate were characterized reactions that required the intake of painkillers and/or significantly reduced the ability of the vaccinee to perform normal daily routine tasks for more than five consecutive hours.

### Antibody measurement

The IgG and IgA Abs against the S1 domain of the SARS-COV-2 spike (S) protein were detected in both serum and swab samples using the respective EUROIMMUN anti-SARS-CoV-2 ELISA assays (EUROIMMUN, Lübeck, Germany, IgA: EI 2606-9601 A and IgG: EI 2606-9601 G). For serum samples the assay was performed according to manufacturer’s instructions and as previously described (13). OD ratios were calculated based on the sample and calibrator OD values. For all analytes, an OD ratio of ≥1.1 was considered as positive. Serum samples with OD ratios >10 were prediluted in sample buffer as previously described (13). For swab samples an in-house customized protocol was used. Both buccal and nasal swabs were left to dry at room temperature in a designated vertical laminar airflow hood. Subsequently, the tips of the swab sticks were inserted in a deep-well plate and 500μl of the sample buffer were pipetted in each well. The plate was then placed on a plate-shaker at medium speed for 20 minutes in order to enhance the release of the sample into the sample buffer. Last, the plate was centrifuged at 3000 rpm for 5 minutes. The eluate of each sample was then pipetted into the assay microplate for final analysis. The eluates were always generated and tested within the same day. The same calibrator and standard samples were used as for the serum samples, therefore OD ratios were calculated accordingly. Again, an OD ratio of ≥1.1 was considered positive.

### Statistical analysis

OD values for ELISA measurements were recorded as numerical continuous variables. Correlation coefficients between the measurements for IgG buccal swab, IgA buccal swab, IgG nasal swab, IgA mucosal swab and the respective measurement in the blood were determined and reported as Pearson correlation coefficient r. Scatterplots between these measurements were created and the respective regression line with 95% confidence interval as well as the regression formula and model diagnostics were added. For cutoff determination for the different measurements in nasal and buccal samples a receiver operating characteristic (ROC) analysis was performed given the result of the blood measurements as reference and the area under the curve (AUC) was computed. In the plots for antibody levels after first and second vaccination, median values with error bars are plotted for different immunization schemes and types of measurement. Statistical significance was set at p<0.05.

## Results

### Cohort characteristics

Overall, 69 healthy adult individuals with a median age of 55 years (range 18-65y) were enrolled in the study. The majority of the subjects were female (76.8%). All participants received a full vaccination scheme according to the then current guidelines with BNT162b2, mRNA-1273, ChAdOx1-nCoV-19 or a combination of those. According to vaccination scheme (booster dose not included) the participants were categorized in overall seven groups. The majority received a double dose of BNT162b2 (44.9%), while all but 4 participants that initially received ChAdOx1-nCoV-19 were subsequently vaccinated with either BNT162b2 or mRNA-1273. Three subjects with positive COVID-19 anamnestic were only vaccinated once according to then applied guidelines. The intervals between first and second dose varied according to the vaccine administered, with 3 to 4 weeks being the standard interval for the mRNA vaccines and 6-12 weeks for ChAdOx1-nCoV-19. Data regarding side-effect profile of the various vaccination schemes, detectability of mucosal anti-S IgA and IgG Abs as well as information on booster dose and breakthrough infection are summarized in **Table 1**. It is of note that information regarding the last two parameters is based on follow-up up to mid-April 2022. Although no sequencing of the causative variant was performed, based on epidemiological data at the time of infection, it is safe to assume that one of the highly immune-evasive Omicron SARS-CoV-2 variant must have caused all breakthrough infections after the booster dose (15).

**Table 1:** Cohort characteristics according to vaccination scheme.

	BNT162b2, BNT162b2	mRNA-1273, mRNA-1273	ChAdOx1, ChAdOx1	ChAdOx1, BNT162b2	ChAdOx1, mRNA-1273	mRNA-1273, NA*	ChAdOx1, NA*	Total
Number of vaccines	31 (44.9%)	13 (18.8%)	4 (5.8%)	11 (15.9%)	7 (10.1%)	2 (2.9%)	1 (1.6%)	69 (100%)
Median age (years)	50	53	60	55	39	55	58	55
female	24 (77.4%)	8 (61.5%)	3 (75%)	9 (81.8%)	7 (100%)	2 (100%)	0	53 (76.8%)
male	7 (22.6%)	5 (38.5%)	1 (25%)	2 (18.2%)	0	0	1 (100%)	16 (23.2%)
Previous SARS-COV-2 infection	0	2 (15.4%)	0	0	0	2 (100%)	1 (100%)	5 (7.2%)
No side-effects after 1st dose	22 (71%)	5 (41.7%)	0	0	1 (14.3%)	0	1 (100%)	29 (42.6%)
Mild side-effects after 1st dose	9 (29%)	5 (41.7%)	2 (50%)	4 (36.4%)	1 (14.3%)	1 (50%)	0	22 (32.4%)
Moderate side-effects after 1st dose	0	2 (16.6%)	2 (50%)	7 (63.6%)	5 (71.4%)	1 (50%)	0	17 (25%)
No side effects after 2nd dose	10 (32.3%)	1 (8.3%)	2 (50%)	4 (36.4%)	0	n.a.	n.a.	17 (26.2%)
Mild side-effects after 2nd dose	16 (51.6%)	6 (50%)	2 (50%)	6 (54.5%)	5 (71.4%)	n.a.	n.a.	35 (53.8%)
Moderate side-effects after 2nd dose	5 (16.1%)	5 (41.7%)	0	1 (9.1%)	2 (28.6%)	n.a.	n.a.	13 (20%)
Missing data	0	1 (7.7%)	0	0	0	0	0	1 (1.4%)
Positive IgG Abs in serum after 1st dose	19† (95%)	11§ (100%)	4 (100%)	10 (90.9%)	7 (100%)	2 (100%)	1 (100%)	54†§ (96.4%)
Positive IgG Abs in buccal swabs after 1st dose	7† (35%)	4§ (36.4%)	0	0	1 (14.3%)	2 (100%)	1 (100%)	17†§ (30.4%)
Positive IgG Abs in nasal swabs after 1st dose	11† (55%)	10§ (90.9%)	0	1 (9.1%)	3 (42.9%)	2 (100%)	1 (100%)	30†§ (53.6%)
Positive IgA Abs in serum after 1st dose	17† (85%)	11§ (100%)	2 (50%)	4 (36.4%)	4 (57.1%)	2 (100%)	1 (100%)	41†§ (73.2%)
Positive IgA Abs in buccal swabs after 1st dose	0†‡	6§ (54.5%)	1 (25%)	0	1‡ (16.7%)	2 (100%)	1 (100%)	13†‡§ (23.6%)
Positive IgA Abs in nasal swabs after 1st dose	15†‡ (78.9%)	10§ (90.9%)	3 (75%)	3 (37.3%)	5‡ (83.3%)	2 (100%)	1 (100%)	41†‡§ (74.5%)
Positive IgA Abs in serum after 2nd dose	31 (100%)	11§ (100%)	4 (100%)	11 (100%)	7 (100%)	n.a.	n.a.	64*§ (100%)
Positive IgA Abs in buccal swabs after 2nd dose	23 (74.2%)	11§ (100%)	0	8 (72.7%)	7 (100%)	n.a.	n.a.	51*§ (79.7%)
Positive IgA Abs in nasal swabs after 2nd dose	27 (87.1%)	11§ (100%)	0	7 (63.6%)	7 (100%)	n.a.	n.a.	54*§ (84.4%)
Positive IgA Abs in serum after 2nd dose	30 (96.8%)	11§ (100%)	1 (25%)	10 (90.9%)	7 (100%)	n.a.	n.a.	59*§ (92.2%)
Positive IgA Abs in buccal swabs after 2nd dose	12 (38.7%)	10§ (90.9%)	1 (25%)	3 (27.3%)	3 (42.9%)	n.a.	n.a.	31*§ (48.4%)
Positive IgA Abs in nasal swabs after 2nd dose	27 (87.1%)	11§ (100%)	1 (25%)	8 (72.7%)	6 (85.8%)	n.a.	n.a.	55*§ (85.9%)
Booster with BNT162b2	10 (32.3%)	3 (23.1%)	3 (75%)	6 (54.5%)	1 (14.3%)	0	0	42 (60.9%)
Booster with mRNA-1273	19 (61.3%)	8 (61.5%)	1 (25%)	5 (45.5%)	6 (85.8%)	2 (100%)	1 (100%)	42 (60.9%)
Breakthrough infection after 2nd dose	2 (6.5%)	0	1 (25%)	0	1 (14.3%)	0	0	4 (5.8%)
Breakthrough infection after 3rd dose	8 (25.8%)	1 (7.7%)	1 (25%)	1 (9.1%)	2 (28.6%)		0	13 (18.8%)

* Due previous infection only one dose was administered.

† For 11 vaccinees in the BNT162b2, BNT162b2 group no sample was available after first dose and before second.

‡ For four vaccines in the BNT162b2, BNT162b2 group and one in the ChAdOx1, mRNA-1273 group igA Abs were not tested in swab samples after first dose.

§ The two convalescent participants from this group were excluded from this analysis. They had detectable Abs in all samples tested percentages are calculated with respect to the sum of valid samples in each category.

### Mucosal IgG and IgA anti-S Abs detectable in majority of vaccinees after second vaccination dose

Although first vaccination dose was not able to induce a detectable mucosal humoral immunity in a significant number of participants - yet with marked differences observed between different vaccination groups (see **Table 1**) -, second dose led to clear detection of IgG and/or IgA Abs in mouth and/or nose in >81% of the subjects. In serum, IgG Abs were detectable in 94.8% and 100% of subjects after first and second vaccination dose, respectively. The corresponding rates for IgA anti-S Abs were 70.7% after first and 93.9% after second vaccine dose. The positivity detection rates per vaccination group are summarized in **Table 1**. It should be noted that for 11 vaccinees in the BNT162b2, BNT162b2 group no sample was available after first and before second dose for testing. Additionally, in five overall vaccinees no test for IgA Abs was performed in the respective swab samples. Those cases were accordingly removed from the applicable analyses. Last, the samples from the two convalescent participants in the mRNA-1273, mRNA-1273 group were also removed from this descriptive statistical analysis.

### IgG anti-S Abs from buccal swab samples associate more consistently with those measured in serum

**Figure 1:**
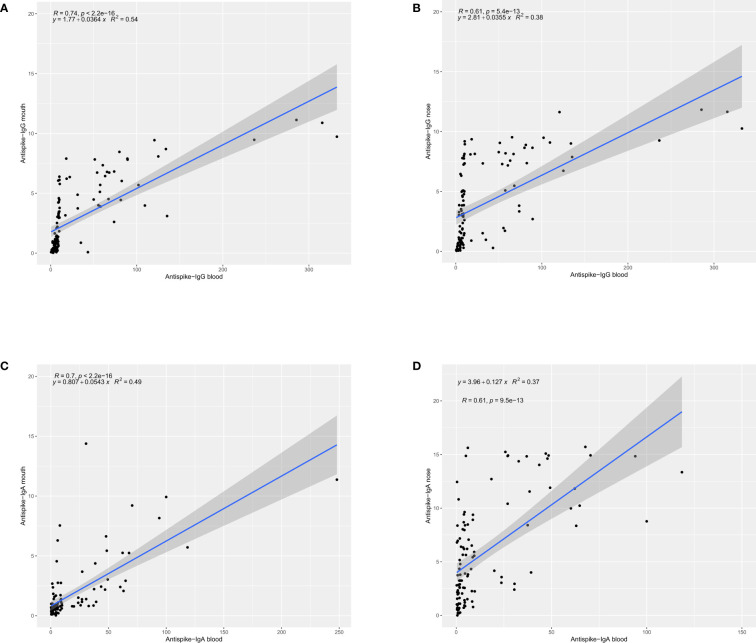
Scatterplot graphical display of correlation of the ELISA OD measurements for IgG and IgA Abs in buccal and nasal swabs with the respective measurements in blood. Correlation was determined and reported as Pearson correlation coefficient r. **(A)** Correlation between IgG OD measurement in buccal swabs and blood, **(B)** Correlation between IgG OD measurement in nasal swabs and blood, **(C)** Correlation between IgA OD measurement in buccal swabs and blood, **(D)** Correlation between IgA OD measurement in nasal swabs and blood.

One of the main objectives of this study was to establish a reliable and consistent method for IgG and IgA anti-S Ab detection in samples from buccal and nasal mucosal surfaces. To this end, OD ratios from buccal and nasal samples were compared with those from serum. Statistical analysis of these data yielded a relatively good correlation coefficient of r=0.74 for the IgG assay in buccal swabs. For nasal swabs correlation was inferior with an r=0.61. The IgA assay exhibited higher divergence and lower concordance, especially in nasal swab samples, for which the correlation coefficient was only 0.61. These results are graphically depicted in **Figures 1A–D**.

**Figure 2:**
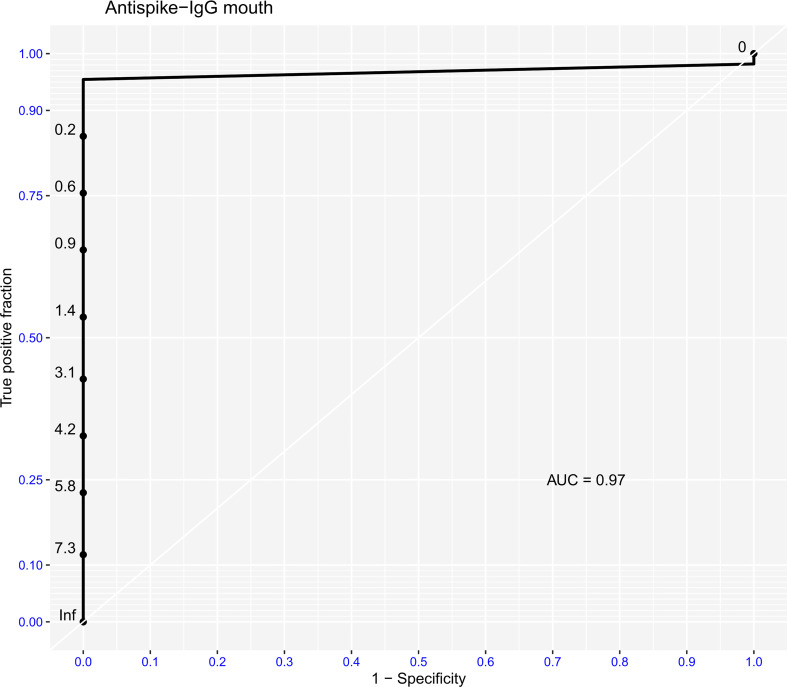
Graphical display of ROC analysis for optimal cutoff determination of the buccal swab assay with serum results serving as reference. The values of the y axis in black represent the respective OD cutoff values. The outer scale in blue represents the sensitivity while the values of the x axis in blue represent the 1-specificity with value 0.0 accounting for 100% specificity. The AUC was computed on account of the ROC analysis.

Given that the IgG assay in buccal swabs demonstrated the highest correlation with serum samples, we proceeded in establishing an adjusted cut-off in order to achieve best possible accuracy, which would render the assay suitable for qualitative immunity screening. Indeed, reduction of OD ratio cut-off from 1.1 to 0.2 for the IgG buccal swab assay led to a sensitivity of 91.8% and specificity of 100%. This calculation was based on overall 520 paired measurements. The result is also graphically presented in **Figure 2**.

### Higher concentration of IgA anti-S Abs at nasal mucosal surface

IgA anti-S Abs were markedly more prevalent in samples from the nasal compared to the buccal mucosa. It is of note, that in 69.5% of the participants IgA anti-S Abs were detectable in nasal swabs although the corresponding serum sample had an OD ratio under the cut-off. This finding could be indicative of either cross-reactive IgA Abs present on nasal mucosa or higher degree of contamination with blood in nasal swab samples compared to the buccal ones.

### mRNA vaccination and previous SARS-CoV-2 infection associate with higher anti-S Abs OD ratios in blood and mucosal surfaces

**Figure 3:**
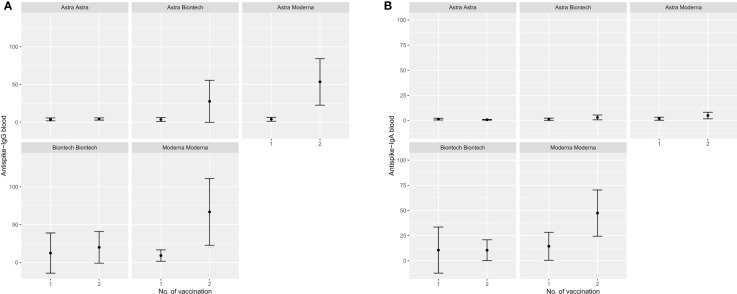
Error bar plots depicting the median OD values reached anti-S Abs in blood after first and second vaccination with different vaccination schemes. **(A)** IgG anti-S Abs in blood, **(B)** IgA anti-S Abs in blood.

**Figure 4:**
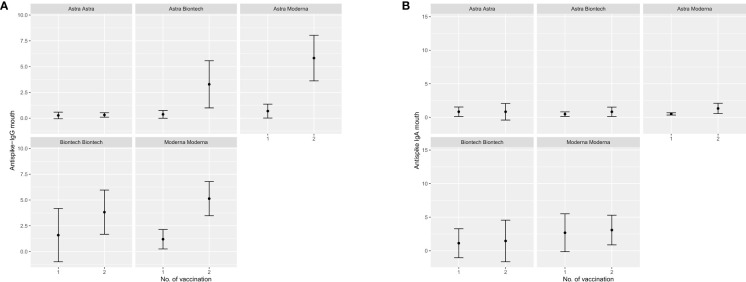
Error bar plots depicting the median OD values reached anti-S Abs in buccal swans after first and second vaccination with different vaccination schemes. **(A)** IgG anti-S Abs in buccal swabs, **(B)** IgA anti-S Abs in buccal swabs.

**Figure 5:**
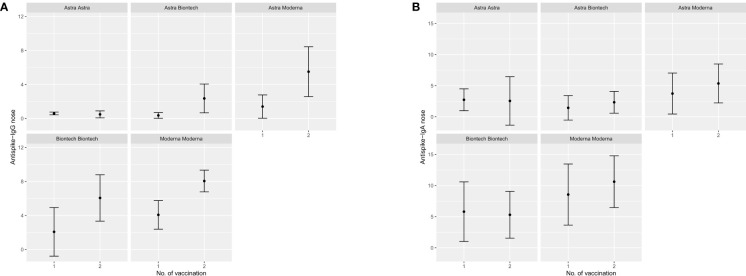
Error bar plots depicting the median OD values reached anti-S Abs in nasal swans after first and second vaccination with different vaccination schemes. **(A)** IgG anti-S Abs in nasal swabs, **(B)** IgA anti-S Abs in nasal swabs.

Participants that were either vaccinated at least once with an mRNA vaccine or were convalescent for SARS-CoV-2 before first vaccination exhibited significantly higher mean IgG and IgA anti-S Abs OD ratios compared to their peers that received only ChAdOx1-nCoV-19. Further comparison between BNT162b2 only, mRNA-1273 only or combination of these two with ChAdOx1-nCoV-19 indicates a higher potency of mRNA-1273 to induce a robust humoral immunity systemically as well as at mucosal surfaces compared to BNT162b2. These results are depicted in the form of errorbar plots for IgG and IgA Abs mean OD ratio after first and second vaccination in **Figures 3**–**5**. Percentages of detectable Abs in both serum and mucosae per vaccination group are also presented in detail in **Table 1**.

## Discussion

Mucosal immunity has long been established as one of the main lines of defense especially against pathogens that use certain mucosae as route of entry into the human body (10). Despite this commonly accepted fact, most research around the efficacy of the newly developed vaccines against SARS-CoV-2 focused primarily on their ability to induce a robust systemic humoral and T cell mediated immunity (7, 16). Two and a half years after the enrollment of mass-scale vaccinations worldwide, we still have limited data regarding the efficiency of different SARS-CoV-2 vaccines to confer a protective mucosal humoral immunity (16–20). Furthermore, immunity screening *via* standard blood-assays can be both logistically and financially unaffordable when applied in large populations. Lately, an increasing number of studies focus on the development of Ab detection from mucosal samples (14, 16–22), particularly after the development of swab collection kits that are able to yield higher amounts of sample from mucosal surfaces. Taken these two issues into consideration we designed a study, which had as main objectives primarily the development of a reliable and sufficiently accurate method for IgG and IgA anti-S Ab detection in samples from the buccal and nasal mucosae and secondarily the comparison of different vaccination schemes as to their ability to induce a detectable mucosal humoral immunity. To our knowledge, this is the first study, which reports a comparative assessment of induced mucosal immunity from both buccal and nasal mucosae with respect to different vaccination schemes with BNT162b2, mRNA-1273, ChAdOx1-nCoV-19 or a combination of them.

Here we show that it is feasible to detect IgG and IgA anti-S-specific antibodies using flocked swabs for sample collection from buccal and nasal mucosae in a slightly modified ELISA assay designed for detection in serum samples. The IgG assay for buccal swabs exhibited the highest consistency as to correlation with serum samples. Although samples from the nasal mucosa showed overall higher sensitivity, the correlation coefficient to serum samples was lower. This is most probably due to the frequent contamination of these samples with blood spots as nasal mucosa is more susceptible to capillary bleeding upon sample collection than the buccal one. The mucosal samples were eluted in sample buffer and were subsequently tested without further dilution. A precise estimation as to the concentration ratio between serum and mucosal samples is not possible, given that our assay protocol offered only a semi-quantitative estimation of the detected Abs. However, considering that serum samples were always diluted at a 1:101 ratio, it is plausible to assume that the concentration of IgG Abs on buccal and nasal mucosae must be about two orders of magnitude lower than that in serum, as already reported by others previously (16). Although very specific, our buccal swab assay did not initially meet the sensitivity threshold of 90% that would render it acceptable for immunity screening (22). Nevertheless, when the applied cut-off was reduced to 0.2 from 1.1, the assay reached a 91.8% sensitivity without any reduction in the already achieved specificity of 100%. Other study groups have also reported assay protocols with similarly good accuracy (21, 22) but to the best of our knowledge we are the first to achieve this with a very simple and inexpensive assay protocol. The main advantage of our assay is that no special sample collection kit with extra fluid supplement is required in order to prohibit immunoglobulin degradation by saliva proteases as the flocked swabs dry fast ensuring preservation of the IgG Abs even months after initial sample collection (14). This can be of vital importance when samples cannot be processed shortly after collection and logistical resources are limited.

As far as IgA anti-S Abs are concerned, the correlation with serum samples appeared to be much less consistent. This was markedly more prominent in the nasal swab samples with 69.5% of subjects having detectable IgA Abs in nasal samples although the respective OD ratio in serum was below the cut-off. This finding may reflect the prevalence of pre-existing cross-reactive secretory Abs against the SARS-CoV-2 spike 1 subunit as this has already been observed and reported previously (23). Possible admixture of blood in the swab samples probably acts more as an enhancer rather than sole factor causing this discordance. Guirrieri et al. also reported data on IgA anti-S Abs in nasal secretions after vaccination or infection (20). Although they used the same assay for IgA anti-S detection, their protocol differed significantly from ours with regard to sample collection and preparation. Furthermore, no direct correlation of OD ratios between serum and nasal samples was sought in that study. With respect to our results in buccal samples, they seem to be in line with those reported by Azzi et al. indicating that the actively induced humoral immunity after vaccination at the buccal mucosa as reflected by IgA anti-S Abs is rather limited (16). Divergent data from other study groups highlight the necessity for standardization of the methods used for Ab detection from mucosal surfaces (19, 24, 25). What also remains to be further elucidated, is to what extent these IgA Abs observed in the nasal swab samples are the result of active production induced by the intramuscularly administered vaccines or are more the reflection of past infections (e.g. from other coronaviridae) having promoted cross-reactive specificities (23–27).

Another important objective of this study was to check for differences in elicited systemic and mucosal humoral immunity between different vaccine regimens. The initial plan was to analyze three overall vaccination schemes as it was assumed that the respective vaccines would be administered as initially authorized. However, an increasing number of reported deaths especially in younger ages after administration of ChAdOx1-nCoV-19 led to a whole new vaccination landscape with combinations becoming the new norm for initially ChAdOx1-nCoV-19 vaccinees (28). This development had as a result an unbalanced distribution of participants in the respective subgroups with very low numbers in some of them. Yet, although limited to descriptive statistics, our findings do add some new information as until now mucosal immunity after vaccination for SARS-CoV-2 has only been sporadically investigated with most reports concerning mainly the BNT162b2 mRNA vaccine (16–20, 24). The superiority of mRNA-1273 in eliciting a robust systemic humoral and T cell mediated immunity has been indicated by an increasing number of study reports (13, 29–31). What is less clear is whether this also translates into an equally superior induction of mucosal immunity compared to its peers. Our results appear to support this notion as participants vaccinated with mRNA-1273 had the highest probability to have detectable Abs on mucosal surfaces compared to non-convalescent participants receiving other vaccines. Furthermore, the mean OD ratios reached were also higher for these participants. Subjects who received mRNA-1273 as second dose in a heterologous vaccination scheme after ChAdOx1-nCoV-19 also exhibited higher frequency of Ab detection and higher mean OD ratios in the swab samples compared to those who received BNT162b2 (32). The higher dosage of the mRNA-1273 compared to that of BNT162b2 (i.e. 100 μg vs 30 μg) is the most commonly accepted explanation for this difference in performance (29–31). Also in line with previously reported data, non-convalescent participants in our cohort who only received ChAdOx1-nCoV-19 had overall the lowest probability of Ab detection in mucosal samples while they also exhibited the lowest mean OD ratios at all analyses (13, 33). The mean OD ratios of convalescent subjects were also markedly higher compared to those of participants with negative COVID-19 anamnestic. These results are also in line with previous reports (13, 16, 34–36). As to the side-effect profile of the different vaccine schemes, our findings again confirm previously published data which indicated a higher frequency of mild to moderate side-effects after first vaccination with ChAdOx1-nCoV-19 (13, 37, 38). Subjects receiving the mRNA-1273 also reported more often mild to moderate side-effects especially after the second dose compared to those who received the BNT162b2 (39). This finding further supports a dose-dependent effect being responsible for the differences observed between these two mRNA vaccines. Last, in our cohort we recorded until mid-April 2022 an overall 24.6% frequency of breakthrough infections with more than 76% of them occurring after the administration of the booster dose highlighting the decreased efficacy of the current vaccines to contain the spread of the recently emerged and highly immune-evasive Omicron variant (15). It is of note that all breakthrough infections had a mild disease course with none of them requiring hospitalization or prolonged isolation.

Our study has certain limitations. One of them is undoubtedly the low number of subjects in the respective vaccination subgroups, which precluded statistical inference testing for comparisons. Moreover, a third vaccination was not considered as certain subjects had either not yet received a third vaccination or did not return for follow-up after it. Also, the time-interval between second and third vaccination showed a wide range in study participants that were evaluated after third vaccination. Due to this high degree of divergence, measurements after third vaccination were excluded from this analysis. Furthermore, we were not able to standardize an assay for determination of Ab neutralizing activity in mucosal samples, although the significance of these Abs as exclusive surrogate marker of protection against infection has been questioned lately (40), especially viewed under the prism of constant emergence of new variants with immune-evasive mutations at the receptor binding domain of the spike protein. We used a commercial kit for antibody detection, that was originally designed for blood samples. If in the future kits for nasal or buccal swabs are developed, it would be useful to include an internal control for assessment of sample collection quality.

## Conclusion

In conclusion, this is the first study to date attempting a comparative assessment of induced mucosal immunity after different vaccination schemes for SARS-CoV-2 by analyzing the prevalence of IgG and IgA Abs in samples from both the buccal and nasal mucosae. Our findings confirm a weaker yet clear prevalence of Abs in mucosal surfaces after full vaccination for SARS-CoV-2 yet with markedly higher IgA Abs prevalence in the nasal cavity, while they also insinuate a relative superiority of the mRNA-1273 to elicit these Abs. Last, our proposed method for IgG Ab detection in buccal swabs has the potential to serve as a reliable alternative to the standard serum assay especially in the context of immunity screening of large populations. It may be interesting to compare measurements from antibody induction by natural infection with antibodies induced by vaccination in the future.

## Data availability statement

The original contributions presented in the study are included in the article/supplementary materials. Further inquiries can be directed to the corresponding author.

## Ethics statement

The Ethical Committee of the University of Ulm (Nr. 488/20) approved the study. The patients/participants provided their written informed consent to participate in this study.

## Author contributions

CT, BJ, HS, and DaF are principal investigators. They designed the study, performed data analysis/interpretation and wrote the manuscript. CL, JS, MP, JH performed ELISA testing; IR, SK, DoF, CN, and EA reviewed the data and edited the manuscript. All authors contributed to the article and approved the submitted version.

## Funding

This work received grant support from the Ministry for Science, Research and Arts of Baden-Württemberg, Germany, and the European Commission (HORIZON2020 Project SUPPORT-E, 101015756) to HS and from the German Red Cross Blood Transfusion Service Baden-Württemberg – Hessen to BJ.

## Conflict of interest

The authors declare that the research was conducted in the absence of any commercial or financial relationships that could be construed as a potential conflict of interest.

## Publisher’s note

All claims expressed in this article are solely those of the authors and do not necessarily represent those of their affiliated organizations, or those of the publisher, the editors and the reviewers. Any product that may be evaluated in this article, or claim that may be made by its manufacturer, is not guaranteed or endorsed by the publisher.
